# Male tarsi specific odorant-binding proteins in the diving beetle *Cybister japonicus* sharp

**DOI:** 10.1038/srep31848

**Published:** 2016-08-22

**Authors:** Li-Mei Song, Xiang Jiang, Xue-Min Wang, Jin-Dong Li, Fang Zhu, Xiong-Bing Tu, Ze-Hua Zhang, Li-Ping Ban

**Affiliations:** 1 College of Animal Science and Technology, China Agricultural University, Beijing, 100193, China; 2 HuangPu Entry-Exit Inspection and Quarantine Bureau, Guangdong, 510730, China; 3 Institute of Animal Science, Chinese Academy of Agricultural Sciences, Beijing, 10 0193, China; 4 Department of Entomology, Washington State University, Pullman, WA, 99164, USA; 5 Institute of Plant Protection, Chinese Academy of Agricultural Sciences, Beijing, 100193, China

## Abstract

Odorant binding proteins (OBPs) play critical roles in chemical communication of insects, as they recognize and transport environmental chemical signals to receptors. The diving beetle *Cybister japonicus* Sharp shows a remarkable sexual dimorphism. The foreleg tarsi of males are equipped with large suction cups, believed to help holding the female during underwater courtship and mating. Here, we identified two OBPs highly and specifically expressed in male tarsi, suggesting important functions of these structures in chemical communication. The first protein, *Cjap*OBP1, exhibits the 6 conserved cysteines motif of classic OBPs, while the second, *Cjap*OBP2, contains only four cysteines and can be assigned to the sub-class of C-minus OBPs. Both proteins were expressed in a bacterial system and the purified recombinant proteins were used to for antibodies preparation. Western Blot analysis showed that *Cjap*OBP1 is predominantly expressed in male tarsi and could be also detected in antennae and palpi of both sexes, while *Cjap*OBP2, besides male tarsi, is also present in testis. Ligand-binding experiments showed a good binding affinity between *Cjap*OBP1, *Cjap*OBP2 and citral and coniferyl aldehyde, respectively. These results support a possible function of these two OBPs in the male foreleg tarsi of diving beetles in chemical communication.

Chemoreception in insects is mediated by membrane-bound receptors^1^,^2^,^3^,^4^,^5^ and soluble proteins, named as odorant-binding proteins^6^,^7^,^8^ and chemosensory proteins^9^,^10^,^11^,^12^. In particular, odorant-binding proteins (OBPs) are small proteins made of α-helical domains and folded into a compact structure^13^,^14^ further stabilized by three interlocked disulfide bridges^15^,^16^. Although six conserved cysteines are the landmark of classical OBPs, members with a lower or higher number of cysteines have been reported in all insect orders and are referred to as ‘Minus-C’, ‘Plus-C’ OBPs, respectively. In addition, atypical members with a different arrangement of the cysteines or with additional domains have also been described^17^,^18^,^19^.

OBPs are abundantly expressed in the lymph of chemosensilla, between the cuticle and the dendritic membrane of sensory neurons^20^,^21^,^22^. The role of OBPs in olfactory processing is generally referred to as that of solubiliser and carrier of hydrophobic pheromones and odorants. Several recent studies have demonstrated that OBPs are also involved in detection and discrimination of semiochemicals^23^,^24^,^25^,^26^,^27^,^28^,^29^. Moreover, functional studies with receptors expressed in heterologous systems have reported that the presence of the appropriate OBPs increases the sensitivity and selectivity of the receptors to pheromones^30^,^31^,^32^,^33^. However, their specific function in sensory organs of insects and in particular the interplay between OBPs and olfactory receptors remain largely unknown.

Besides chemosensory organs, OBPs are also abundantly expressed in secretory glands and cells that often produce species specific pheromones. In such organs, OBPs are believed to help release of such semiochemicals into the environment. Several studies have been reported, in the pheromone glands of *Bombyx mori*^34^ and *Agrotis ipsilon*^35^, in the seminal fluid of *Aedes aegypti*^36^ and *Helicoverpa* species^37^, as well as in the mandibular glands of *Apis mellifera*^38^. Some other OBPs are also involved in roles unrelated to chemoreception. For example, the OBP56a expressed in the oral disk of the blow fly *Phormia regina* was suggested to be a fatty acid solubiliser^39^. In *Aedes aegypti*, several OBPs were identified as components of the egg shell^40^,^41^, suggesting their functions beyond chemoreception.

Up to date, many OBPs have been identified in several species of Coleoptera, the largest order in the class of Insecta, including *Anomala osakana*^42^, *Popillia japonica*^42^ and *Phyllopertha diversa*^43^, and more recently, in *Tenebrio molitor*^44^, *Tribolium castaneum*^45^, *Anoplophora glabripennis*^46^, *Dendroctonus valens*^47^, *Dendroctonus ponderosae* (obtained with second generation deep sequencing) and *Lissorhoptrus oryzophilus* (Curculionoidea). However, information regarding olfactory proteins remains scarce in aquatic beetles.

The diving beetle *Cybister japonicus* Sharp (Coleoptera: Dytiscidae) lives in stagnant waters^48^,^49^ and is part of an important group of predatory insects. The beetles of the Dytiscidae family often show a range of peculiar secondary sexual characters in both males and females. The three basal segments of the pro-tarsi in males are usually equipped with various combinations of small and large suction cups. These cups are suggested to play roles in climbing, swimming and catching preys, as well as reproduction^50^,^51^,^52^. In such context, males use specialized proleg tarsi to adhere the slippery females elytra^50^.

In this study, we reported the first two OBPs in the diving beetle *C. japonicas*. The structure modeling, specialized expression in male tarsi and ligand-binding properties of these OBPs suggested their potential function in chemical communication between male and female diving beetles.

## Results

Aim of the present work is to elucidate the function of the giant male front tarsi in water beetles through a study of the proteins expressed, focusing on odorant-binding proteins. The clear sexual dimorphism and the use of these anatomical structures in courtship and mating suggested that they might be involved in chemical communication.

### Morphology of proleg tarsi of *C. japonicus*

**Figure 1:**
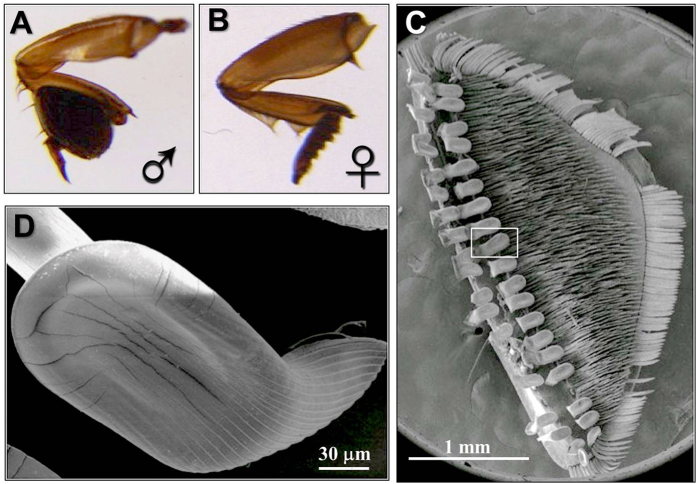
Morphology of proleg tarsi of *C. japonicus*. The proleg of male (**A**) and female (**B**) are sexual dimorphism, in that the prolegs of males are equipped with large suction cups (**C**) absented in females, and bear specialized adhesive setae on their ventral side. Each spatula seta, labeled with the rectangle, is shown at higher magnification in (**D**), and connects to the palette with an off-centre stalk and its ventral surface has an oval-shaped sucker. Scale bar: 1 mm for (**C**), and 30 µm for (**D**).

Although the peculiar structure of male tarsi in water beetles has been reported^52^, we first undertook a morphological investigation to describe in detail such organ in the diving beetle *C. japonicus* Sharp using scanning electron microscopy (SEM). Figure 1 clearly evidences the remarkable sexual dimorphism of the tarsi. The prolegs of males are equipped with large suction cups, which are absent in females (Fig. 1A,B). The male proleg presents an expanded palette composed of protarsomeres bearing specialized adhesive setae on their ventral side (Fig. 1A). Each spatula seta connects to the palette with an off-centre stalk and its ventral surface has an oval-shaped sucker from which parallel channels extend distally (Fig. 1C,D). The structural elements of this organ are very similar to those reported for the male diving beetle *Cybister rugosus*^50^ and proposed to control female movement.

### Identification of odorant-binding proteins

**Figure 2:**
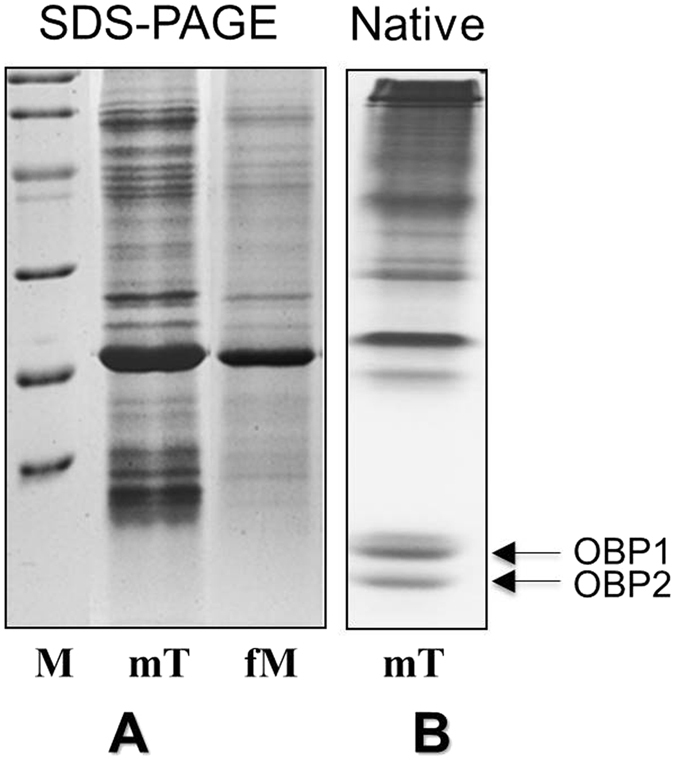
Expression of *Cjap*OBP1 and *Cjap*OBP2 from the tarsi of male *C. japonicus*. (**A**) SDS-PAGE analysis of tatsi from both male and female showed the abundant presence of low molecular weight bands mainly in the crude extracts from the tarsi of male *C. japonicus*. (**B**) A crude extract of male tarsi was separated on a native gel, and two fast migrating bands were selected for N-terminal sequencing. mT, male tarsi; fT, female tarsi. Molecular mass markers (M) are (from the top): 97 kDa (phosphorylase B), 66 kDa (BSA), 45 kDa (ovalbumin), 29 kDa (carbonic anhydrase), 20 kDa (trypsin inhibitor) and 14 kDa (α-lactalbumin).

We hypothesized that the exaggerated growth of male tarsi could be related to semiochemical delivery. Therefore we decided to investigate the protein composition of the tarsi in a comparative way between male and female diving beetles. Electrophoretic analysis (SDS-PAGE) of crude extracts from the tarsi of male and female *C. japonicus* showed the presence of low molecular weight bands mainly in the male sample (Fig. 2A). Given the unusual abundance of these bands, migrating with apparent molecular masses compatible with the values expected for OBPs, we decided to investigate their protein composition. Thus, we separated a crude extract of male tarsi on a native gel (Fig. 2B) and selected two fast migrating bands for N-terminal sequencing. Previous work had shown that generally OBPs migrate on the front of native gels and can be easily separated from other proteins^53^. The two selected bands were blotted onto PVDF membrane and subjected to N-terminal sequencing. We obtained the N-terminal 20 amino acids sequences LDDAQKAKFKAHYDLCVTET and ISPEQKEKMKKLHDECLHET for these two proteins and temporary named them as protein 1 and protein 2.

### Molecular Cloning, sequence and analysis and structural modeling of *Cjap*OBPs

The information on the first 20 amino acids enabled us to design degenerate primers (Supplementary Table S1) at the 5′ ends of two genes. These degenerated primers together with oligo-(dT)_18_V at the 3′ end were used to amplify the relative genes with PCR. 5′-RACE was then performed to complete the sequences at the 5′ end, so we obtained the full-length genes, as reported in the Materials and Methods section.

On the basis of their significant similarities with OBPs from other species in National Center for Biotechnology Information (NCBI) database, the two deduced protein sequences were named *Cjap*OBP1 and *Cjap*OBP2 and deposited in the GenBank database with accession numbers KX078770 and KX078771, respectively.

**Figure 3:**
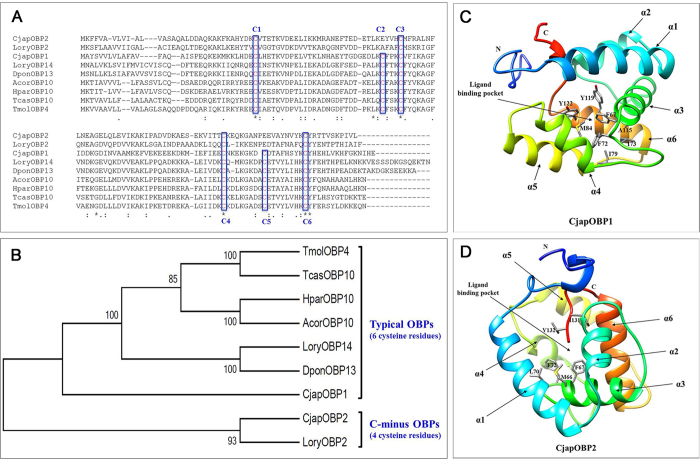
Structure of *Cjap*OBPs and phylogenetic relationship of *Cjap*OBPs with other beetle OBPs. (**A**) The newly identified *Cjap*OBP1 (a typical OBP with six cysteines) and *Cjap*OBP2 (a C-minus OBP with only four cysteines) are aligned with seven OBP proteins from other Coleoptera. The asterisks indicate the conserved amino acids. The conserved cysteine residues are highlighted in blue box. The phylogenic tree (**B**) was constructed with amino acid sequences of *Cjap*OBP1 and *Cjap*OBP2 as well as seven OBP proteins from other beetles using MEGA 6 software. The tertiary structures of CjapOBP1 (**C**) and CjapOBP2 (**D**) were stimulated based on the selected protein templates from I-TASSER.

*Cjap*OBP1 is a single polypeptide of 138 amino acids, with a calculated molecular weight for the mature protein of 14.1 kDa and a predicted isoelectric point of 5.49. *Cjap*OBP2 is similar in length, with 132 amino acids, a calculated molecular weight of 13.6 kDa and an isoelectric point of 6.74 for the mature protein. While *Cjap*OBP1 presents the six-cysteine signature of classic insect OBPs, *Cjap*OBP2 contains only four cysteines and can be assigned to the family of C-minus OBPs. The sequences of the two OBPs of *C. japonicus* were aligned with seven representative OBPs from other Coleoptera species (Fig. 3A). Phylogenetic analysis was performed based on amino acid sequences alignment of these beetle OBPs. The phylogenetic tree was generated by MEGA 6.06 with the neighbor-joining algorithm. The nine beetle OBPs separated into two groups, one group with six cysteine residues and the other having four cysteine residues (Fig. 3B). The overall structures of *Cjap*OBP1 and 2 (predicted by I-TASSER with highest ranking templates being 3qme and 3d77, respectively) consist of six helices (labelled α1 to α6) surrounding the hydrophobic ligand binding pocket (Fig. 3C,D). The predicted ligand binding site residues for *Cjap*OBP1 include seven hydrophobic amino acids (Phe67, Phe72, Ile73, Ile79, Met84, Tyr119 and Tyr122) (Fig. 3C). The putative ligand binding site residues for *Cjap*OBP2 consist of six hydrophobic amino acids (Met66, Phe67, Leu70, Phe72, Ile131 and Val132) (Fig. 3D).

### Specific localization of *Cjap*OBPs in male proleg tarsi

Using pET28a as a vector, we obtained the proteins fused with a His-tag fragment in high yields and in their soluble forms. Purification was performed by affinity chromatography on Ni-NTA His-Bind column. The electrophoresis analyses relative to expression and purification steps were reported in Supplementary Figure S1.

**Figure 4:**
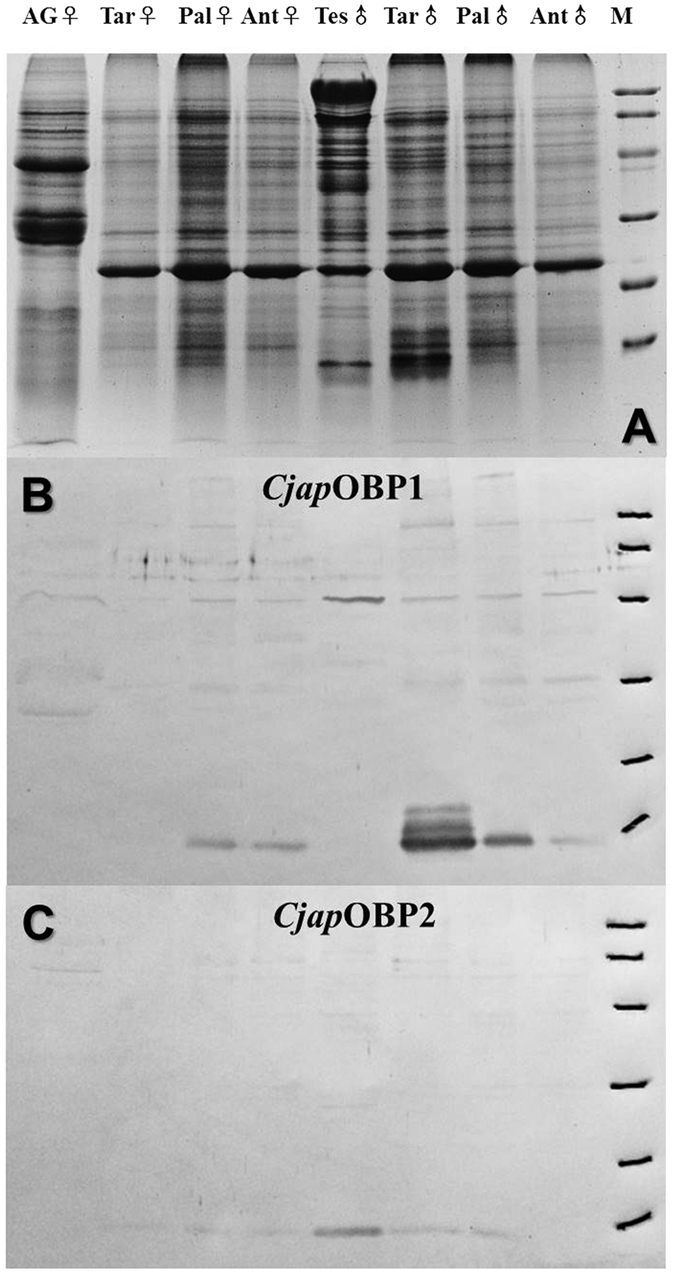
SDS-PAGE and Western blot of crude extracts from different parts of the body of adult *C.japonicus*. (**A**) SDS-PAGE analysis of crude extracts from different tissues of male and female *C. japonicas*, showed the presence of low molecular weight bands mainly in the male tarsi sample. Western blot membrane after reaction with polyclonal antiserum against *Cjap*OBP1 (**B**) or *Cjap*OBP2 (**C**) and peroxidase-conjugated second antibody. Ant, antennae; Tar, tarsi; Pal, palpi; AG, accessory gland; and Tes, testis. Molecular mass markers (M) are (from the top): 97 kDa (phosphorylase B), 66 kDa (BSA), 45 kDa (ovalbumin), 29 kDa (carbonic anhydrase), 20 kDa (trypsin inhibitor) and 14 kDa (α-lactalbumin).

Western blot experiments, using a polyclonal antiserum raised against the purified protein, revealed that *Cjap*OBP1 is very abundant in male tarsi, with relatively low expression in palpi and antennae of both sexes (Fig. 4). *Cjap*OBP2, instead was detected in testis and tarsi of males (Fig. 4). Both proteins, therefore appear to be mainly expressed in the tarsi with a strong sex bias, suggesting their potential functions in chemical communication between male and female diving beetles.

### Fluorescence binding assays

**Figure 5:**
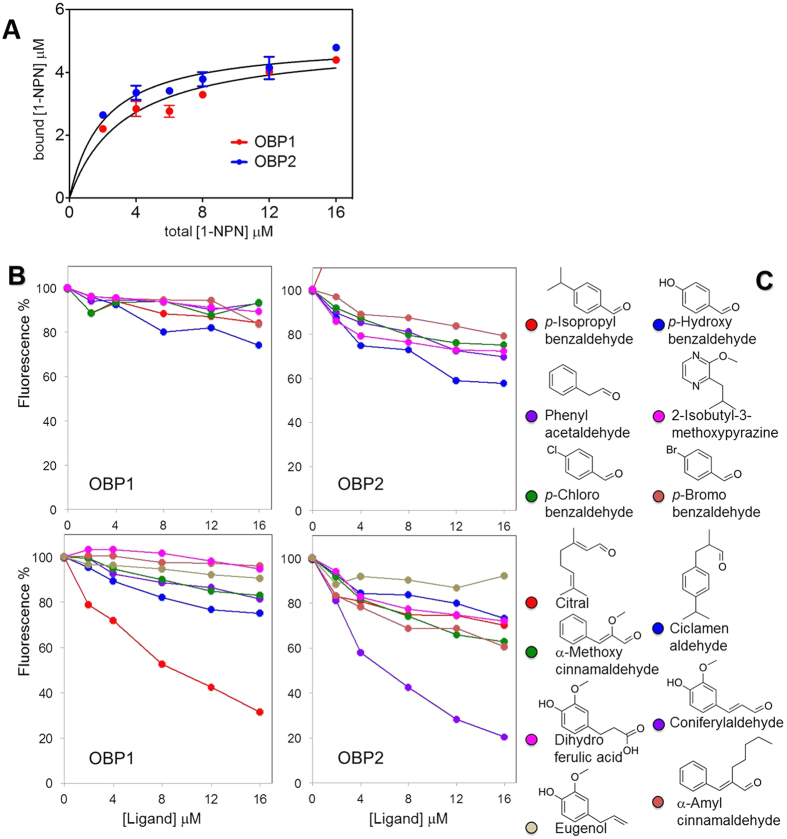
Affinity of *Cjap*OBPs to candidate chemical ligands. (**A**) Both *Cjap*OBP1 and *Cjap*OBP2 bind the fluorescent probe 1-NPN, with dissociation constants of 3.3 μM and 2.1 μM, respectively. (**B**) Competitive binding curves of selected ligands to the two *Cjap*OBPs. Mixtures of proteins (5 μM) and 1-NPN (2 μM) were titrated with 1mM solutions of the ligands in methanol. Among the ligands tested, *Cjap*OBP1 shows good affinity only to citral, and *Cjap*OBP2 bind coniferyl aldehyde strongly, but not dihydroferulic acid, eugenol and α-methoxycinnamaldehyde, which are structurally related to coniferyl aldehyde. (**C**) The chemical structures of the chemicals applied.

To characterize the ligand-binding properties of the two OBPs, we performed fluorescence binding assays with several chemicals having different structures using N-phenyl-1-naphthylamine (1-NPN) as a fluorescent reporter. In the absence of any information about potential semiochemicals for this insect, as well as for related species, we selected some common plant volatiles, such as citral, coniferyl aldehyde, eugenol and 2-isobutyl-3-mthoxypyrazine, and structurally similar compounds. 1-NPN binds *Cjap*OBP1 and CjapOBP2 with dissociation constants of 3.3 and 2.1 μM respectively (Fig. 5A). Among the ligands tested, we identified a single good ligand for each protein, citral for *Cjap*OBP1 and coniferyl aldehyde for *Cjap*OBP2 (Fig. 5B,C). In particular, we can observe that several compounds structurally related to coniferyl aldehyde, such as dihydroferulic acid, eugenol and α-methoxycinnamaldehyde, did not show appreciable binding to *Cjap*OBP2, indicating that this protein might have a rather narrow binding structure spectrum.

## Discussion

In this study, we have identified two OBPs predominantly expressed in the frontal tarsi of male *C. japonicus*. The deduced amino acid sequences suggested that *Cjap*OBP1 consists of a typical framework of OBPs (six-conserved cysteines), while *Cjap*OBP2 contains only four cysteines and belongs to the family of C-minus OBPs (Fig. 3). Both proteins present high sequence similarities with OBPs of other Coleoptera. The first Coleoptera insect OBPs were identified from scarab beetles, specifically expressed in antennae, and associated with the pheromone recognition based on ligand-binding properties and single sensillum recordings evidences^42^. With the advantage of next-generation sequencing, a growing number of OBPs have also been reported in other Coleoptera species^54^,^55^,^56^,^57^,^58^. The diversified functions of these OBPs might be revealed in the future with future exploration.

*C.japonicus* has marked sexual dimorphism of the tarsi with SEM investigation (Fig. 1). It has been known that males use their giant front tarsi to hold on the back of females during their courtship^50^,^51^,^52^. The high expression of OBPs in the specialized front tarsi of males may suggest significant roles interfering with sex communication. Because during the underwater courtship the female *C. japonicus* could release sex pheromones used by males for partner localization that was observed in the diving beetle *Rhantus suturalis*^59^. Therefore it is reasonable to hypothesize that these OBPs specific expressed in male foreleg tarsi *C. japonicus* may be involved in the sex pheromone reception. Work is in progress in our lab to determine the functions of *Cjap*OBP1 and *Cjap*OBP2 through functional RNA interference and behavior studies. In swallowtail bufferfly *Papilio xuthus*, several chemosensory proteins and OBPs were identified in the female foreleg tarsi, which is served as a chemosensory organ in this species, suggesting an important function of these proteins in oviposition behavior of *P. xuthus*^60^. Most recently, two putative OBPs show abundant expression in palps and foreleg tarsi of *Amblyomma americanum*, which contain the olfactory Haller’s organ. These *A. americanum* OBPs may also play roles in sex pheromones reception^61^. Future functional studies of these foreleg tarsi abundant OBPs will shed new lights on the understanding of molecular basis and evolution of chemoreception.

We can speculate that such proteins, rather than being involved in chemosensing, act as solubilisers and carriers of pheromones, and OBPs are transferred to females during such process together with their load of pheromones, as reported in several other species^12^.

Investigating the binding affinity and distribution of expression of OBPs is useful for understanding the physiological function and mechanism of olfactory recognition^37^,^62^. The fluorescent binding experiments showed that each protein is coupled with a single good ligand, citral for *Cjap*OBP1 and coniferyl for *Cjap*OBP2. The components of the sex pheromones in these beetles are unknown, but we can hypothesize that molecules structurally similar to citral and coniferyl aldehyde might represent good candidates, on the basis of our preliminary binding experiments.

## Materials and Methods

### Ethics statement

The use of rabbits in our experiments has been approved by the Institutional Animal Care and Use Committee of China Agricultural University (permit number: SYXK 2007–0023). All experimental protocols and procedures were carried out according to relevant regulations and guidelines established by this committee. All efforts were made to minimize the suffering of the rabbits.

### Insect collection and rearing

Diving beetle *Cybister japonicus* Sharp were obtained from Guangdong Province, China, and raised in the Grassland Science Department (China Agricultural University, Beijing, China) at 28–30 °C, with photoperiod of 18 h:6 h (light: dark). The aquariums were filled with tap water, and the water was renewed twice a week. The aquariums were covered with fly screen to prevent beetles from flying away.

### Scanning electron microscopy

For scanning electron microscopy (SEM), the samples of male prolegs were fixed in 70% ethanol for 2 h, and then cleaned in ultrasonic bath (250 W) for 1 min in the same solution. After treatment with 100% ethanol for 30 min, the samples were dried in air. Prolegs of male were mounted on holders, and after gold-coating, the samples were examined in a FEI Quanta 200 SEM (FEI Company, the Netherlands).

### Identification of *C. japonicus* OBPs

The different parts of the *C.japonicus* including antennae (30 pairs), tarsi (10 for male, and 30 for female), palpi (30 pairs), and sex organs (3) of both sexes were collected on ice and ground under liquid nitrogen. The samples were extracted with 0.3–1.0 mL 50 mM PBS (pH: 7.4), containing 1% PMSF and centrifuged at 13000 rpm for 20 min. Supernatants were collected and analyzed by 15% SDS-PAGE. For sequencing analysis, a sample of male tarsi was separated on a 15% native gel and blotted onto PVDF membrane. Fast migrating bands were subjected to automated Edman degradation, using a Procise 492 protein sequencer (Applied Biosystems, Foster City, Calif.), equipped with a 140C microgradient apparatus and a 785A UV detector (Applied Biosystems, Foster City, Calif.) for the automated identification of phenylthiohydantoin-amino acids.

### RNA extraction, cDNA synthesis, and cloning

Total RNA was extracted using the TRIzol^®^ Reagent (Sigma, USA), following the manufacturer’s procedure. cDNA was synthesized with total RNA and Reverse Transcript PCR kit (Qiagen, Germany), along with the protocol provided. The PCR products were amplified using degenerated primers designed based on the amino acid residues of the N-terminal sequencing and oligo-(dT)_18_V primer (Supplementary Table S1). After a step at 50 °C for 30 min and 95 °C for 15 min, the reaction was performed for 35 cycles (95 °C for 0.5 min, 47 °C for 0.5 min, 72 °C for 1 min), followed by a final step of 7 min at 72 °C.

Crude PCR products (both about 500 bp) were then ligated into a pGEM-T (Promega, USA) vector. After transformation of *Escherichia coli* XL-1 Blue competent cells with the ligation product, positive colonies were selected by PCR using the plasmid’s primers SP6 and T7, then extracted and sequenced. Analysis of five colonies for each sample gave the same sequences.

### 5′ RACE-PCR of putative *Cjap*OBP gene fragements

Reverse transcription and 5′ rapid amplification of cDNA ends (RACE) were carried out with the SMART-RACE cDNA Amplification Kit (Clontech, USA). Total RNA from the front tarsi of male beetle was used as a template for first-strand synthesis with a 5′-CDS primer A and SMARTer II A oligo according to manufacturer’s directions. Second-strand synthesis was carried out with the PCR kit (Tiangen, China) with the 5′ RACE outer primer UPM (Clontech, USA) and specific forward primer. The primers used were shown in Supplementary Table S1. The 20 μl reaction mixture was initially incubated at 5 cycles of 94 °C for 30 s, 72 °C for 3 min, followed by 5 cycles of 94 °C for 30 s, 70 °C for 30 s, and 72 °C for 3 min, and end with 27 cycles of 94 °C for 30 s, 68 °C for 30 s, and 72 °C for 3 min. The single reaction product was gel isolated and cloned in pGEM-T (Promega, USA). Eight positive clones for each sample were sequenced.

### *In silico* structural analysis

Amino acid sequences of *Cjap*OBPs and other beetle OBPs, including: *Lory*OBP2 (KF383281.1) and *Lory*OBP14 (KF383275.1) from *Lissorhoptrus oryzophilus*; *Tmol*OBP4 (KP071918.1) from *Tenebrio molitor*; *Tcas*OBP10 (XM_970591.2) from *Tribolium castaneum*; *Acor*OBP10 (KM251650.1) from *Anomala corpulenta*; *Hpar*OBP10 (KR733556.1) from *Holotrichia parallela*; *Dpon*OBP13 (KP736119.1) from *Dendroctonus ponderosae* were alignment using MUSCLE (http://www.ebi.ac.uk/Tools/msa/muscle/). The secondary and tertiary structures of *Cjap*OBP1 and *Cjap*OBP2 were predicted by an online protein structure homology modeling server I-TASSER (http://zhanglab.ccmb.med.umich.edu/I-TASSER/). Then the Protein Data Bank (PDB) coordinate file of the highest ranking model was loaded into Chimera (http://www.cgl.ucsf.edu/chimera) for molecular visualization and modification.

### Phylogenetic tree construction

The insect OBP amino acid sequences were analyzed using MUSCLE alignment default settings through MEGA version 6.06^63^ (http://www.megasoftware.net/). The phylogenic tree was then generated by the neighbor-joining algorithm since the p-distance was <0.8 in overall distance^64^. A total of 2,000 bootstrap replications were used to test of phylogeny. Finally, the selected tree was created with the cut-off value of 50%.

### Heterologous expression and purification of recombinant *Cjap*OBP proteins

pGEM plasmids containing sequences of the mature *Cjap*OBPs were digested with NdeI and BamHI restriction enzymes. Then the products were ligated into a pET28a vector (Novagen, Germany) linearised with the same enzymes. The recombinant pET-*Cjap*OBP expression plasmid was transformed into *E. coli* BL21 (DE3) competent cells. Single colonies were grown overnight in 4 ml of Luria-Bertani (LB) broth supplemented with 50 μg/ml kanamycin at 37 °C with shaking at 200 rpm. The culture was diluted 1:100 with fresh LB broth (supplemented with 50 μg/ml kanamycin) and grown at 37 °C with shaking at 200 rpm until the OD_600_ reached approximately 0.5. Isopropyl β-D-1-thiogalactopyranoside (IPTG) (Merck, Germany) was then added to the culture to a final concentration of 0.4 mM to induce expression of the target products for approximately 3 h at 37 °C. After sonication of the bacterial pellet and centrifugation, all proteins were present in the supernatant, and the recombinant *Cjap*OBP protein were purified using a Ni-NTA His-Bind column following the provided protocol. (Novagen, Germany).

### Preparation of antisera

Polyclonal antisera against *Cjap*OBP1 and *Cjap*OBP2 were obtained by injecting rabbits subcutaneously and intramuscularly with 500 μg of purified recombinant protein, followed by three additional injections of 300 μg after 10 days each time. The protein was emulsified with an equal volume of Freund’s complete adjuvant (Sigma, St. Louis, MO, USA) for the first injection and incomplete adjuvant (Sigma, St. Louis, MO, USA) for further injections. The blood of rabbits was taken 7 days after the last injection, and centrifuged at 6000 rpm for 30 min. The supernatant of the serum was used without further purification. Rabbits were individually housed in large cages, at constant temperature, and all operations were performed according to ethical guidelines in order to minimize pain and discomfort to animals. The immunizations were performed according to the protocol approved^65^,^66^.

### Western blot analysis

Extracts were prepared from antennae, mouth parts, tarsi, sex organs of adults of both sexes, as described in the Section of “Identification of *C. japonicus* OBPs” in “Materials and Methods”. After electrophoretic separation, gels were electroblotted onto nitrocellulose (NC) membrane (Millipore, USA) using a semi-dry protocol^67^. Following overnight treatment with 2% powdered skimmed milk, the membrane was incubated with the crude antiserum at a dilution of 1:500 (2 h) and then with Goat Anti-Rabbit IgG (H+L), Horseradish Peroxidase Conjugate (Invitrogen, USA) (a dilution of 1:1000 for 1 h). Immunoreacting bands were detected by treatment with 4-chloro-1-naphthol and hydrogen peroxide.

### Binding assays

To measure the affinity of the fluorescent ligand N-phenyl-1-naphthylamine (1-NPN), a 2 μM solution of the protein in 50 mM Tris-HCl (pH 7.4) was titrated with aliquots of 1 mM 1-NPN in methanol to final concentrations of 1–16 μM. The probe was excited at 337 nm, and emission spectra were recorded between 380 and 450 nm. The affinity of other ligands was measured in competitive binding assays, using the protein and the fluorescent reporter 1-NPN at the concentration of 5 μM and 2 μM respectively, while increasing the final concentration of each competitor up to 16 μM. Dissociation constants for 1-NPN were evaluated from Scatchard plots of the binding data, and other ligands were calculated from the corresponding IC_50_ values, using the equation: K_D_ = [IC_50_]/1+[1-NPN]/K_1-NPN_, [1-NPN] being the free concentration of 1-NPN and K_1-NPN_ being the dissociation constant of the complex protein/1-NPN.

## Additional Information

**How to cite this article**: Song, L.-M. *et al*. Male tarsi specific odorant-binding proteins in the diving beetle *Cybister japonicus* sharp. *Sci. Rep*. **6**, 31848; doi: 10.1038/srep31848 (2016).

## Supplementary Material

Supplementary Information
