# Supplementary material for: Male tarsi specific odorant-binding proteins in the diving beetle Cybister japonicus sharp
Source: Sci Rep. 2016 Aug 22;6:31848. doi: 10.1038/srep31848 (PMC4992826; doi:10.1038/srep31848)
Supplement: Supplementary Information [file srep31848-s1.pdf]

Male tarsi specific odorant-binding proteins in the diving beetle *Cybister japonicus* sharp

Li-Mei Song[1, +], Xiang Jiang[2, +], Xue-MinWang[3], Jin-Dong Li[1], Fang Zhu[4], Xiong-Bing Tu[5], Ze-Hua Zhang[5], Li-Ping Ban[1, *]

[1] College of Animal Science and Technology, China Agricultural University, Beijing, 100193, China

[2] HuangPu Entry-Exit Inspection and Quarantine Bureau, Guangdong, 510730, China

[3] Institute of Animal Science, Chinese Academy of Agricultural Sciences, Beijing, 10 0193, China

[4] Department of Entomology, Washington State University, Pullman, WA, 99164, USA

[5] Institute of Plant Protection, Chinese Academy of Agricultural Sciences, Beijing, China, 100193

[+] These authors contributed equally to this work.

* Corresponding author

E-mail: liping_ban@163.com

Figure S1. Bacterial expression and purification of *Cjap*OBPs.

Both *Cjap*OBP1(A) and *Cjap*OBP2（C）were obtained in high yields (about 30 mg /L of culture) in its soluble form and purified by Ni-NTA His-Bind column. The left panels (A, C) report the SDS-PAGE analysis relative to crude bacterial extracts before (Pre) and after (Ind) induction with IPTG, plus the supernatant (Sup) and the pellet (Pellet) after the sonication. The right panels (B, D) show the purification steps of the two proteins, with 3 fractions for *Cjap*OBP1 and 5 fractions for *Cjap*OBP2 from the last purification step. Molecular weight markers (M) are as in Figure 4.

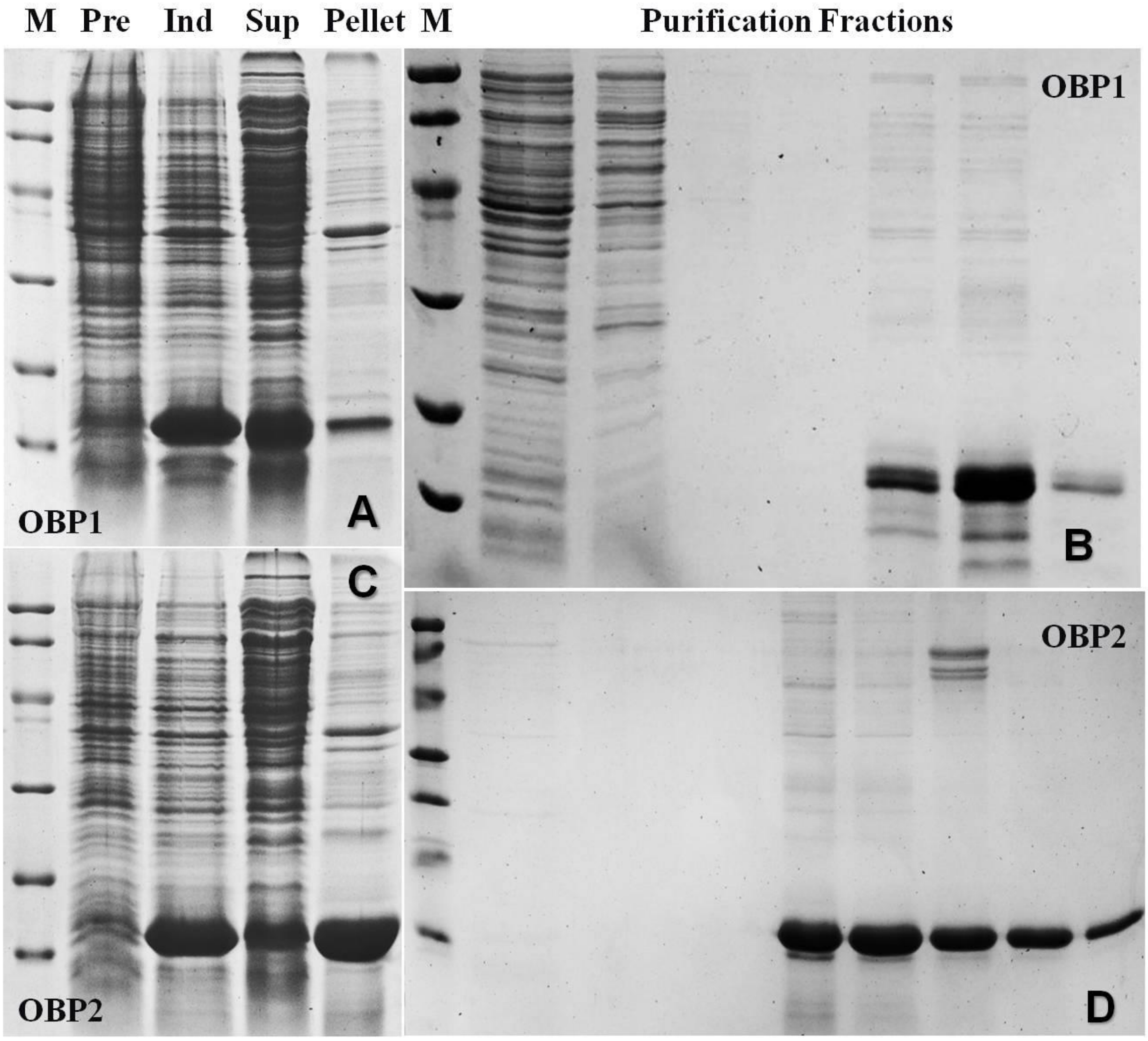

Table S1. Sequences of all primers used.

RT-PCR primers for amplification of *Cjap*OBP1 and *Cjap*OBP2

CjapOBP1-fw: GARCARAARGARAARATGAA

CjapOBP1-rv: TTTTTTTTTTTTTTTTTTV

CjapOBP2-fw:CARAARGCNAARTTYAARGC

CjapOBP2-rv: TTTTTTTTTTTTTTTTTTV

Primers for 5′ **RACE of *Cjap*OBP1 and *Cjap*OBP2**

CjapOBP1-fw: GTCGTCTATGAAGCCTGCCTTGA

CjapOBP2-fw: CTCGGTGAATTCGTTGGCGCGCA

Primers for expression of the mature *Cjap*OBPs in pET28a (NdeI and BamHI restriction sites are underlined)

CjapOBP1-fw: CATATGATATCACCCGAGCAAAAAG

CjapOBP1-rv: GGATCCTTATTCATGGATGTTTTTGCC

CjapOBP2-fw: CATATGCAGGCTCTCGATGATGCCCA

CjapOBP2-rv: GGATCCTTAAAGAACAATAGGTTTGCG
